# Microbial Diversity of Source and Point-of-Use Water in Rural Haiti – A Pyrosequencing-Based Metagenomic Survey

**DOI:** 10.1371/journal.pone.0167353

**Published:** 2016-12-09

**Authors:** Nabanita Mukherjee, Debra Bartelli, Cyril Patra, Bhavin V. Chauhan, Scot E. Dowd, Pratik Banerjee

**Affiliations:** 1 Division of Epidemiology, Biostatistics, and Environmental Health, School of Public Health, University of Memphis, Desoto Avenue, Memphis, Tennessee, United States of America; 2 Molecular Research LP (MR DNA), Shallowater, Texas, United States of America; Adjunct Professor, UNITED STATES

## Abstract

Haiti endures the poorest water and sanitation infrastructure in the Western Hemisphere, where waterborne diseases cause significant morbidity and mortality. Most of these diseases are reported to be caused by waterborne pathogens. In this study, we examined the overall bacterial diversity of selected source and point-of-use water from rural areas in Central Plateau, Haiti using pyrosequencing of 16s rRNA genes. Taxonomic composition of water samples revealed an abundance of Firmicutes phyla, followed by Proteobacteria and Bacteroidetes. A total of 38 bacterial families and 60 genera were identified. The presence of several *Klebsiella* spp. (tentatively, *K*. *pneumoniae*, *K*. *variicola* and other *Klebsiella* spp.) was detected in most water samples. Several other human pathogens such as *Aeromonas*, *Bacillus*, *Clostridium*, and *Yersinia* constituted significantly higher proportion of bacterial communities in the point-of-use water samples compared to source water. Bacterial genera traditionally associated with biofilm formation, such as *Chryseobacterium*, *Fusobacterium*, *Prevotella*, *Pseudomonas* were found in the point-of-use waters obtained from water filters or domestic water storage containers. Although the pyrosequencing method utilized in this study did not reveal the viability status of these pathogens, the abundance of genetic footprints of the pathogens in water samples indicate the probable risk of bacterial transmission to humans. Therefore, the importance of appropriate handling, purification, and treatment of the source water needed to be clearly communicated to the communities in rural Haiti to ensure the water is safe for their daily use and intake.

## Introduction

Access to safe water is a serious public health concern in Haiti, the nation with the poorest water and sanitation infrastructure in the Western Hemisphere [1]. The World Health Organization (WHO) estimates only 62% of Haitians have access to “improved water” sources [2]. The situation is even worse for those who live in rural areas; only 48% of Haiti’s rural population has access to such sources of water compared to 85% of the urban population [3]. Therefore, according to the World Bank’s most recent population data, an estimated 2.4 million people in rural Haiti lack access to improved water sources [4]. The WHO/UNICEF Joint Monitoring Programme (JMP) for Water Supply and Sanitation defines an improved water source as one that “*by the nature of its construction or through active intervention*, *is protected from outside contamination*, *in particular from contamination with faecal matter*” [5]. Categories of improved drinking water sources include piped water, public taps, boreholes or tubewells, protected dug wells, protected (capped) springs and rainwater. Taking into consideration the fact that improved drinking water is not necessarily safe water [6, 7] the proportion of the country’s rural population at an elevated risk of poor health due to exposure to contaminated drinking water is probably much higher than estimates indicate. In fact, the majority of those living in the countryside must rely on drinking untreated or unimproved water at least part of the time [1, 7].

Waterborne diseases, mostly infectious in nature, caused several thousand deaths in Haiti in recent years [1]. It is further estimated that the diseases spread by contaminated water are major causes of death of Haitian children under the age of five [8]. The cholera outbreak in 2010 which claimed more than eight thousand lives underscores the need to understand the overall risk of microbial contaminants transmitted through water [9]. In recent years, the Haitian government’s efforts to improve the situation included the creation of a regulatory body, the National Directorate for Potable Water and Sanitation (DINEPA) which deployed nearly 300 trained Communal Water and Sanitation Technicians (TEPACs) to serve rural communities [10]. Much of the work to improve both water quality and health in Haiti, however, continues to be driven by non-governmental organizations (NGOs) and international agencies [11]. Their approaches include installation of wells, capping springs and introduction of a wide variety of household or point-of-use (POU) water treatment systems. Studies examining the efficacy of these systems indicate the need for ongoing monitoring of both household and source water [6, 8, 11].

To date, most of the studies on the quality of water samples obtained from Haiti dealt with detection of specific organisms (such as *Vibrio cholerae*) using culture-dependent methods involving filtration followed by enrichment and plating or by polymerase chain reaction (PCR) based strategies [12–15]. However, the growth-dependent microbial assays may underestimate the potential presence of other non-target or non-cultivable microorganisms. The advent of the next generation sequencing (NGS) based metagenomics methods enable a broad spectrum identification of different microbes along with a high-resolution visualization of the overall microbiota of a given sample without the need of culturing the bacteria [16, 17]. Studies utilizing NGS techniques to understand the overall microbial composition, ecology, and diversity of different types of environmental media have gained remarkable attention from the scientific communities which is evident by a growing number of reports in recent years [18–21]. Moreover, recent studies indicate that metagenomic tools may identify new set of microbial parameters and targets for a wider, comprehensive, and more representative risk characterization [22, 23]. This eventually will address some key limitations of existing microbial risk assessment of water quality as the current microbial testing methods relying on detection of predetermined microbial indicators are often unable to accurately predict the occurrence of all microbial pathogen resulting in poor risk characterization [22]. Therefore, NGS-based metagenomics may be used effectively to gain in-depth knowledge about the comprehensive microbial quality and safety of the water available to the populations lacking access to improved water sources.

In the current study, we explored the overall bacterial ecology of selected source and POU water samples in a rural area of Haiti’s Central Plateau utilizing culture-independent pyrosequencing of the 16S rRNA genes. Using metagenomics approach, we comprehensively assessed the bacterial diversity associated with the water sources and the POU water used as drinking water by household members living in the Central Plateau area.

## Materials and methods

### Study Area and Source of Water

The study was conducted in a 22 square-mile rural area southwest of Hinche, the regional capital of Haiti’s Central Plateau. The majority of the estimated 20,000 residents of the area are subsistence farmers who speak predominantly Haitian Kreyol. The study area possesses no paved roads and lies 10–35 km from the nearest hospital. The primary sources of water for the community are springs, streams, and rivers. Within the study area, there were no capped springs or other functional improved water sources. Households in the study area utilize one of three types of water filtration systems introduced by a NGO from the United States that has been working in the area for several years. Systems in use include a two-bucket system that employs prechlorination followed by a string filter and granular activated carbon (GAC), and then post-chlorination; slow-sand filtration (biosand); and a 0.10 micron filtration system (Sawyer PointOne™). At the time of data collection, these systems had been in use from 9 years (two-bucket system) to six months (micron filter). The biosand filter was introduced approximately one year prior to the study. Within the study area, it was estimated that 600 households were using the two-bucket system, 20 households had a Sawyer PointOne™ filter and nine households had a biosand filter. A filtration system is absent in the majority of households in the region.

### Sample Collection and Processing

A total of 116 source and POU water grab samples were collected in 100 mL Security-Snap™ Sterile Coliform Water Sample Bottles (Thermo Fisher, USA) from households and major water sources across eight villages in the study area in June 2015. Sources were the Hinquette and Tablon rivers and their tributaries. All water sources are located in public areas and their use does not require permission. Source locations were identified by household members who accompanied members of the research team and directed them to the location from which they collected household drinking water. Data collection did not involve any endangered or protected species.

Households with biosand and Sawyer PointOne™ filters were identified with the help of trained water technicians residing in the community. A snowball sampling technique was used to identify households with a two-bucket filtration system. Water samples were also taken from neighboring homes that lacked a filtration system. Each water sample location was geocoded using a Garmin eTrex 10 GPS Unit and recorded by the Universal Transverse Mercator (UTM) coordinate system. The geodata were then compiled and analyzed using ArcMap 10.4.1 (Esri Inc. USA) on OpenStreetMap basemap (openstreetmap.org) to produce the maps of water collection sites (S1 Fig). Water samples were stored at refrigeration temperature (4°C) prior to processing by membrane filtration technique using pre-sterilized magnetic filter funnels fitted with sterile hydrophilic polyethersulfone (PES) 47 mm Supor^®^ 200 membrane disc filters with pore size 0.2 μm (Pall Corporation, Port Washington, NY, USA).

### DNA Extraction and Next Generation Sequencing

Metagenomic DNA samples were directly extracted from PES membrane filters using PowerWater^®^ DNA Isolation Kit (MO BIO Laboratories, Carlsbad, CA, USA), following the manufacturer’s protocol, and were quantified spectrophotometrically using a NanoDrop spectrophotometer (Thermo Scientific, Wilmington, DE, USA). For pyrosequencing analyses, DNA from 95 samples was pooled into 18 groups based on the types of major sources (spring, stream or river), filter types, and location (S1 Table).

For metagenomics analyses, we performed bTEFAP® (MR DNA www.mrdnalab.com) described previously [21, 24, 25]. In this study, 16S universal Eubacterial primers 530F (GTGCCAGCMGCNGCGG) and 1100R (GGGTTNCGNTCGTTR) were used to evaluate the microbial ecology of each sample on the 454 GS FLX+ (Roche Diagnostics Corp, Branford, CT, USA) with methods via the bTEFAP® DNA analysis service utilizing the 454 FLX chemistry following manufacturer’s protocols as described in details previously [21, 24, 25].

### Computational and Statistical Analyses

The sequence data with quality score 25 (Q25) were processed using a proprietary analysis pipeline (www.mrdnalab.com, MR DNA, Shallowater, TX, USA) and Operational Taxonomic Units (OTUs) clustering at 3% divergence (97% similarity) were defined as described previously [21, 24, 25]. The taxonomic classification of OTUs were done by using BLASTn against a curated database derived from GreenGenes/RDP/NCBI [26].

For data analyses, different statistical programs were used including “R”, XLstat, NCSS 2007, and NCSS 2010. The overall bacterial diversity (alpha and beta diversity) was analyzed using Quantitative Insights Into Microbial Ecology (QIIME,www.qiime.org) as described previously [24, 25, 27–29]. The differences in bacterial genera between the source and POU water samples were compared using a controlled ANOVA procedure. Significance reported for any analysis is defined as p<0.05.

### Accession Numbers

The metagenomic sequence datasets associated with this paper are archived at NCBI Sequence Read Archive (SRA) under the BioProject accession number of PRJNA322639. BioSample records are accessible with the following accession numbers: SAMN05172269, SAMN05172270, SAMN05172271, SAMN05172272, SAMN05172273, SAMN05172274, SAMN05172275, SAMN05172276, SAMN05172277, SAMN05172278, SAMN05172279, SAMN05172280, SAMN05172281, SAMN05172282, SAMN05172283, and SAMN05172284.

## Results and Discussion

The concentrations of DNA extracted from water samples contained measurable amount of DNA (39–116 ng/μL), while negative controls with no cells had no detectable DNA (data not shown). A total of 116,660 sequences identified after stringent quality sequence curation within the bacterial kingdom. These sequences were utilized for final bacterial microbiota analyses of the eighteen sample groups which were based on the water types sampled (source versus POU samples collected from different villages), including the pooled samples from all water source (rivers or springs) and POU samples. The details of pooling samples and clustering into groups have been described in S1 Table. The average reads per sample was 6481. For alpha and beta diversity analysis, samples were rarefied to 6000 sequences and bootstrapped at 4500 sequences.

**Fig 1:**
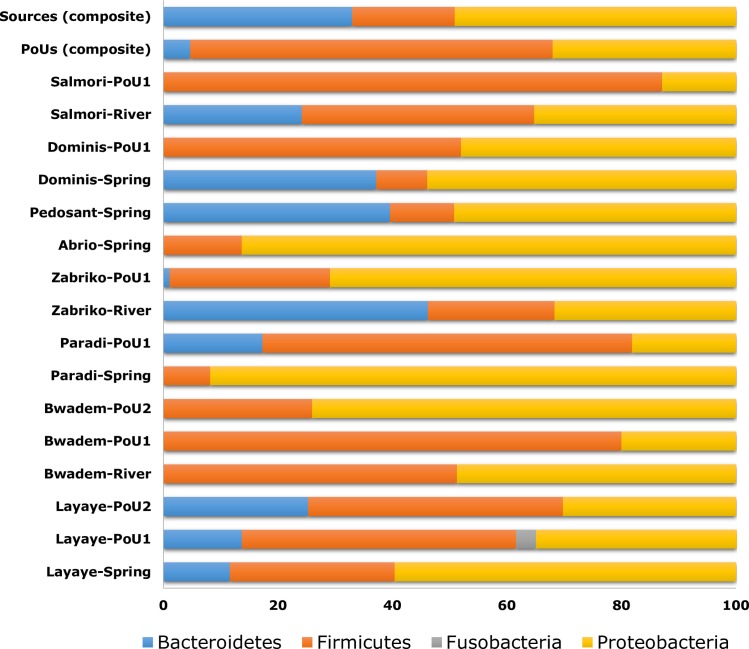
Relative abundance of bacterial diversity in source and point-of-use water samples at phylum level as determined by bTEFAP^®^.

**Fig 2:**
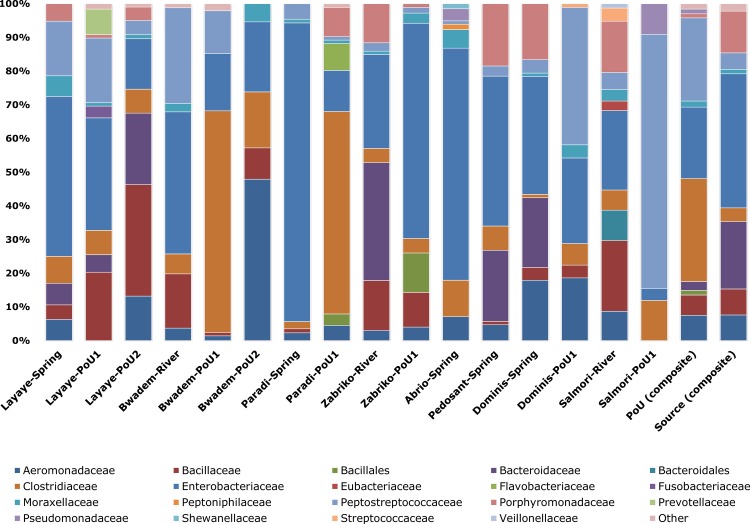
Relative abundance of bacterial families residing in source and point-of-use water samples as determined by bTEFAP^®^. Multi-colored stack bar graphs represent the relative abundance of bacterial family in each sample.

Taxonomical composition revealed that the *Firmicutes* phyla was most common, followed by *Proteobacteria* and *Bacteroidetes* (Fig 1). The least bacterial diversity at the phylum level was found in water samples collected from Abrio and the Paradi spring which consisted mostly of *Proteobacteria* (86.3% and 91.8%, respectively). Prevalence of these phyla have also been reported previously from various types of water samples utilizing both culture-dependent and independent techniques [30–32]. Within these dominant phyla, the bacterial families with the highest relative abundance across most samples were *Aeromonadaceae*, *Bacillaceae*, *Bacillales*, *Bacteroidaceae*, *Bacteroidales*, *Clostridiaceae*, *Enterobacteriaceae*, *Moraxellaceae*, *Peptostreptococcaceae*, and *Porphyromonadaceae* (Fig 2). Among these, *Enterobacteriaceae*, *Bacillaceae* and *Clostridiaceae* are known to include a variety of human pathogenic bacterial species transmittable through environments such as water [33–35].

**Fig 3:**
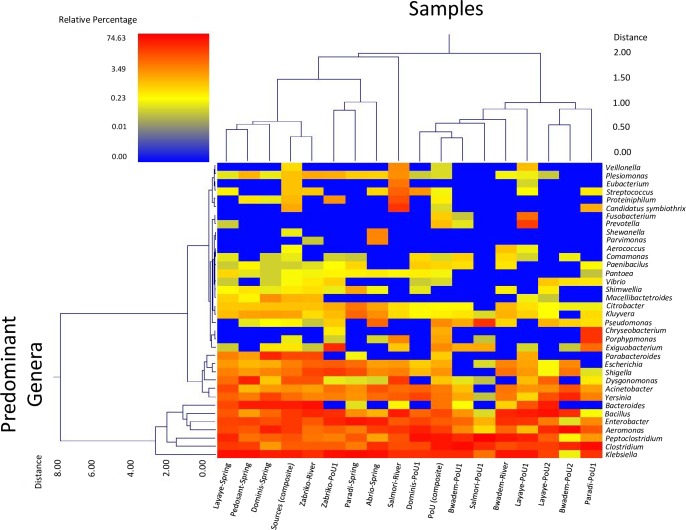
Dual Hierarchal dendrogram evaluation of the taxonomic classification of source and point-of-use water samples. Samples are clustered on the X-axis and labeled based upon the water source types. Samples with more similar microbial populations are mathematically clustered closer together. The genera (consortium) are used for clustering. Thus the samples with more similar consortium of bacterial genera cluster closer together with the length of connecting lines (top of heatmap) related to the similarity, shorter lines between two samples indicate closely matched bacterial consortium. The heatmap represents the relative percentages of each bacterial genus. The predominant genera are represented along the right Y-axis. The legend for the heatmap is provided in the upper left corner.

The relative abundance and bacterial diversity at the genus level is presented in Fig 3. The most prevalent bacterial genus identified across all observed samples was *Klebsiella*. Other bacterial genera such as *Acinetobacter*, *Aeromonas*, *Bacillus*, *Bacteroides*, *Clostridium*, *Dysgonomonas*, *Enterobacter*, *Escherichia*, *Parabacteroides*, *Parvimonas*, *Peptoclostridium*, *Shigella*, and *Yersinia* were commonly found in almost all samples. The genus *Citrobacter* was mainly found in spring water of Paradi (2.6%) but not in samples collected from other locations. The predominant bacterial genera in the water samples analyzed based on the relative abundance cutoff of 1.0% are shown in Figs 3 and S2. A high presence of *Exiguobacterium* was found in the POU water collected from Zabriko (11.7%) and Paradi (3.4%). Bacteria belonging to the genus *Kluyvera* were found mainly in spring water from the villages of Paradi (3.6%), Abrio (1.1%), Pedosant (1.0%), and Dominis (1.0%). *Porphyromonas* was only found in POU water from Paradi (8.6%), and the river water of Salmori (1.6%). *Proteiniphilum* was found in the source water of Salmori (5.9%) and POU water at Zabriko (1%). Both *Pseudomonas* and *Streptococcus* were found in POU as well as in the source water. *Pseudomonas* accounted for 9.1% relative abundance in the POU water samples from Salmori and 3.6% relative abundance in source water from Abrio. *Streptococcus* was found in the river water of Salmori (3.9%) and POU water samples collected from Dominis (1.1%). In addition, our results indicate that some bacterial genera were found exclusively in certain samples, for example, *Chryseobacterium* was only found in POU water from Paradi (8.1%), *Fusobacterium* and *Prevotella* only in POU water samples in Layaye (3.5%, and 7.5%, respectively), *Macellibacteroides* in spring water from Dominis (1.1%), and *Shewanella* in spring water of Abrio (1.5%). Interestingly, some bacteria genus including *Candidatus symbiothrix* (8.8%), *Eubacterium* (2.8%), *Plesiomonas* (1.1%), and *Veillonella* (1.3%) were exclusively found in the river water in the Salmori village.

As indicated above, the most common bacterial genus in both source and POU water samples observed in our study was *Klebsiella* spp (belonging to the bacterial family *Enterobacteriaceae*). Bacteria of the genus *Klebsiella* are a frequent cause of nosocomial infections [36]. We identified the presence of several *Klebsiella* spp. (tentatively, *K*. *pneumoniae*, *K*. *variicola* and other *Klebsiella* spp.) in almost all the drinking water samples collected. Among them, *K*. *pneumoniae* is the most predominant estimated species, followed by *K*. *variicola* and other *Klebsiella* spp. Pathogenic *K*. *pneumoniae* has been associated with urinary tract infections as well as bacteremic liver abscess and can be transmitted easily through person-to-person contact and coming in contact with a contaminated surface especially in a healthcare facility [36–38]. *K*. *pneumoniae* exhibits resistance to several classes of antibiotics, including aminoglycosides, chloramphenicol, fluoroquinolones, tetracycline, and trimethoprim/sulfamethoxazole [39]. One of the most potent antibiotic resistant strains among *Klebsiella* is carbapenem-resistant *K*. *pneumoniae* (CRKP). It has been reported that CRKP is resistant to most of the available antibiotics [40]. Hence the infections caused by CRKP are difficult to treat and thus have been associated with high rates of morbidity and mortality [41]. However, the high prevalence of *Klebsiella* spp. in these samples is not surprising as some of the *Klebsiella* species belong to human normal flora, especially found in skin, mouth and intestine [42]. Presence of enterotoxigenic *Klebsiella* species was reported in populations with gastrointestinal disorders in Haiti in reports published almost four decades ago [14].

Other bacterial species estimated in our study include pathogenic or potentially pathogenic *Aeromonas bestiarum*, *Aeromonas hydrophila*, *Bacillus anthracis*, *Bacillus cereus*, *Dysgonomonas gadei*, *Dysgonomonas capnocytophagoides*, *Enterococcus faecalis*, *Escherichia coli*, *Fusobacterium periodonticum*, *Kluyvera ascorbata*, *Parvimonas micra*, *Plesiomonas shigelloides*, *Porphyromonas spp*., *Prevotella nigrescens*, *Pseudomonas aeruginosa*, *Shewanella putrefaciens*, *Shigella* spp., *Streptococcus oralis*, *Veillonella parvula* and *Yersinia* spp. Additionally, some opportunistic pathogens such as *Acinetobacter baumannii*, *Acinetobacter junii*, and *Citrobacter* spp. were also identified in this study. The diversity of bacterial genera varied significantly between source and POU water (Fig 3). In POU water samples, bacterial genera such as *Aeromonas*, *Bacillus*, *Clostridium*, *Yersinia*, *Exiguobacterium*, *Pseudomonas*, *Porphyromonas*, *Chryseobacterium*, *Prevotella*, and *Fusobacterium* were found to constitute significantly higher potions of the community structure when compared to source waters (Figs 3 and S2). Most of these bacteria are known to transmit from environment (including water, air, soils, and foods) or via human to human pathways and cause various human diseases. For example, pathogenic *Aeromonas* species are ubiquitous in the natural environment and are often food- or waterborne [43, 44]. A prominent member of this genus found in this study, *Aeromonas hydrophila*, can cause a variety of diseases in humans from gastrointestinal disorders to more severe illnesses such as septicemia, meningitis, wound infections, etc. [45, 46]. *Bacillus* spp. (including *B*. *anthracis* and *B*. *cereus*), known to cause both gastrointestinal and non-gastrointestinal infections including severe disease such as anthrax [47, 48], were found extensively in both source and POU samples. Approximately 80% of the samples collected contained *Shigella* spp. and *Yersinia* spp, with high relative abundance in POU water samples. Both of these pathogens may pose a considerable public health threat in less developed countries by causing conditions such as bacillary dysentery, inflammatory autoimmune disorders, and even highly contagious diseases such as plague [49, 50]. Another pathogen *Kluyvera* spp. (*K*. *ascorbata*), found mostly in source water samples (spring or river) in our study, is known to cause urinary tract infection in humans [51, 52]. As revealed by our analysis, high frequency of OTUs related to pathogenic bacteria in POU water may indicate higher risk of human transmission, since these waters are used for drinking.

**Fig 4:**
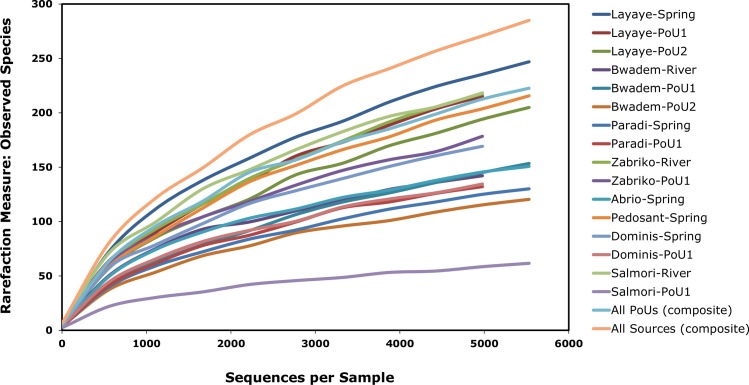
Rarefaction curves showing observed taxonomic units of bacterial species diversity in the source and point-of-use water samples.

**Fig 5:**
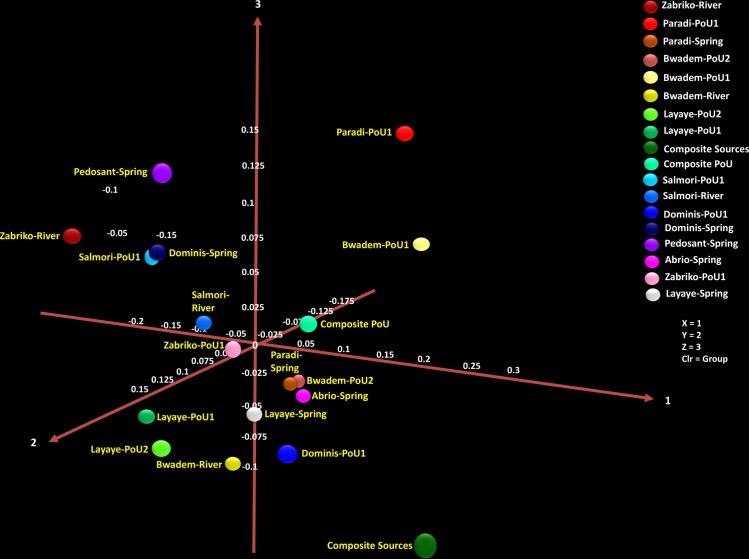
PCoA analysis of the microbiome of each water sample based upon UniFrac method. Different colored symbols are indicative of the different major source and point-of-use water samples. Symbols that are closer together represent similar bacterial community compositions.

For alpha and beta diversity analysis, samples were rarefied to 6000 sequences and bootstrapped at 4500 sequences. A Rarefaction Curve (Fig 4) based on OTUs was constructed to estimate bacterial diversity. A 97% similarity of OTUs as revealed by the Rarefaction Curve modeling at the 3% divergence for each sample suggesting adequate depth of sequencing coverage [21, 24, 25]. Fig 4 reveals that the source water samples from Layaye (spring) were found to contain the highest level of species richness followed by river water samples from Salmori. This could be due, in part, to the location of the spring (adjacent to a river) with the possibility of overrun during heavy rains and cross-contamination caused by near-exclusive use of the spring by local residents. The POU water of Layaye (from two bucket and biosand filters) showed the highest species richness among all the POU samples. The least species richness was observed in POU samples from Bwadem (biosand filter) and Salmori (two bucket system). Using weighted Principal Coordinates Analysis (PCoA) of the microbiome of each sample based upon UniFrac method, we observed no microbiome phylogenetic assemblage relationships among the sample groups. Filtered water samples (two bucket) from Paradi and Bwadem; and source waters from Pedosant (spring) and Zabriko (river) are found to be most distant from the central cluster of other samples (Fig 5). Some samples of different types (source vs POU) and places (geographical location) clustered together, for example, source water samples (spring) from Paradi and Abrio clustered together with POU sample from Bwadem. Similarly, spring water sample from Dominis clustered with filtered (two bucket) samples of Salmori. Therefore, the bacterial community compositions of these samples were independent of the type of place of collection indicating a species-level variability, which is not uncommon and has been attributed to a probable functional redundancy of different or same environmental habitats [53, 54].

Further, we evaluated the impact of water filtration on the composition of autochthonous microbiota of the source water. Filtration devices or certain components of filters (such as sand) may introduce certain species of bacteria associated with biofilm formation (“schmutzdecke”) which may eventually influence the microbial quality of the finished water as reported in previous studies [15, 55, 56]. In our study, we also found depletion or introduction of certain bacterial genera after-filtration in the POU water with reference to the autochthonous microbiota respective water sources (Table 1). It is evident that the bacterial depletion from source waters to the POU water varied across the villages and filters used, but we found no particular trend. For example, in Salmori village, the POU water showed a very high degree of bacterial genera depletion, where 15 out of 18 genera were found to be depleted. While in Zabriko only 3 genera (out of 13 in source river water) were found to be depleted in the POU water. In this context, it is to be noted that DNA markers of bacteria detected in DNA-based methods (such as in metagenomics) can also originate from non-viable cells [57], and therefore, may not be directly associated with presence of viable bacterial cells and the filtration efficiencies of the filters. Interestingly, we detected new genera in the POU water samples (post-filtration) from some villages which were absent in the respective source waters (pre- filtration). All newly introduced genera (Table 1) in POU water samples in our study were reported to include species that are known to be efficient biofilm formers (as referenced here): *Chryseobacterium* [58], *Exiguobacterium* [59], *Fusobacterium* [60], *Porphyromonas* [61], *Prevotella* [62], *Proteiniphilum* [63], *Pseudomonas* [64], and *Streptococcus*[65].

**Table 1:** Taxa depleted or introduced in point-of-use water with respect to corresponding source water.

**Layaye-Spring**			**Layaye-Point-of-Use (filtered, filter type 1)**
**13 Genera**			**Unchanged (9 Genera):** *Acinetobacter*, *Bacillus*, *Bacteroides*, *Clostridium*, *Enterobacter*, *Escherichia*, *Klebsiella*, *Peptoclostridium*, *Shigella*.
*Acinetobacter*, *Aeromonas*, *Bacillus*, *Bacteroides*, *Clostridium*, *Dysgonomonas*, *Enterobacter*, *Escherichia*, *Klebsiella*, *Parabacteroides*, *Peptoclostridium*, *Shigella*, *Yersinia*.* *			**Depleted (4 Genera):** *Aeromonas*, *Dysgonomonas*, *Parabacteroides*, *Yersinia*.
			**Introduced (2 Genera):** *Fusobacterium*, *Prevotella*.
	➞		
			**Layaye-Point-of-Use (filtered, filter type 2)**
			**Unchanged (10 Genera):** *Acinetobacter*, *Aeromonas*, *Bacillus*, *Bacteroides*, *Clostridium*, *Dysgonomonas*, *Enterobacter*, *Klebsiella*, *Peptoclostridium*, *Yersinia*.
			**Depleted (3 Genera):** *Escherichia*, *Parabacteroides*, *Shigella*.
			
**Bwadem-River**			**Bwadem-Point-of-Use (filtered, filter type 1)**
**10 Genera**			**Unchanged (6 Genera):** *Aeromonas*, *Bacillus*, *Clostridium*, *Enterobacter*, *Klebsiella*, *Peptoclostridium*.
*Acinetobacter*, *Aeromonas*, *Bacillus*, *Clostridium*, *Enterobacter*, *Escherichia*, *Klebsiella*, *Peptoclostridium*, *Shigella*, *Yersinia *			**Depleted (4 Genera):** *Acinetobacter*, *Escherichia*, *Shigella*, *Yersinia*.
	➞		
			**Bwadem-Point-of-Use (filtered, filter type 2)**
			**Unchanged (7 Genera):** *Acinetobacter*, *Aeromonas*, *Bacillus*, *Clostridium*, *Escerichia*, *Shigella*, *Yersinia*.
			**Depleted (3 Genera):** *Enterobacter*, *Klebsiella*, *Peptoclostridium*.
			
**Paradi-Spring**			**Paradi-Point-of-Use (filtered)**
**12 Genera**			**Unchanged (7 Genera):** *Acinetobacter*, *Aeromonas*, *Clostridium*, *Enterobacter*, *Klebsiella*, *Peptoclostridium*, *Yersinia*.
*Acinetobacter*, *Aeromonas*, *Bacillus*, *Citrobacter*, *Clostridium*, *Enterobacter*, *Escherichia*, *Klebsiella*, *Kluyvera*, *Peptoclostridium*, *Shigella*, *Yersinia*.* *	➞		**Depleted (5 Genera):** *Bacillus*, *Citrobacter*, *Escherichia*, *Kluyvera*, *Shigella*.
			**Introduced (3 Genera):** *Chryseobacterium*, *Exiguobacterium*, *Porphyromonas*.
			
**Zabriko-River**			**Zabriko-Point-of-Use (filtered)**
**13 Genera**			**Unchanged (10 Genera):** *Acinetobacter*, *Aeromonas*, *Bacillus*, *Clostridium*, *Enterobacter*, *Escherichia*, *Klebsiella*, *Peptoclostridium*, *Shigella*, *Yersinia*.
*Acinetobacter*, *Aeromonas*, *Bacillus*, *Bacteroides*, *Clostridium*, *Dysgonomonas*, *Enterobacter*, *Escherichia*, *Klebsiella*, *Parabacteroides*, *Peptoclostridium*, *Shigella*, *Yersinia*.* *	➞		**Depleted (3 Genera):** *Bacteroides*, *Dysgonomonas*, *Parabacteroides*.
			**Introduced (2 Genera):** *Exiguobacterium*, *Proteiniphilum*.
			
**Dominis-Spring**			**Dominis-Point-of-Use (filtered)**
**13 Genera**	➞		**Unchanged (8 Genera):** *Acinetobacter*, *Aeromonas*, *Bacillus*, *Clostridium*, *Enterobacter*, *Klebsiella*, *Peptoclostridium*, *Yersinia*.
*Acinetobacter*, *Aeromonas*, *Bacillus*, *Bacteroides*, *Clostridium*, *Enterobacter*, *Klebsiella*, *Kluyvera*, *Macellibacteroides*, *Parabacteroides*, *Peptoclostridium*, *Shigella*, *Yersinia*.* *			**Depleted (5 Genera):** *Bacteroides*, *Kluyvera*, *Macellibacteroides*, *Parabacteroides*, *Shigella*.
			**Introduced (1 Genera):** *Streptococcus*.
			
**Salmori-Spring**			**Salmori-Point-of-Use (filtered)**
**18 Genera**			**Unchanged (3 Genera):** *Clostridium*, *Klebsiella*, *Peptoclostridium*.
*Acinetobacter*, *Aeromonas*, *Bacillus*, *Candidatus symbiothrix*, *Clostridium*, *Dysgonomonas*, *Enterobacter*, *Escherichia*, *Eubacterium*, *Klebsiella*, *Peptoclostridium*, *Plesiomonas*, *Porphyromonas*, *Proteiniphilum*, *Shigella*, *Streptococcus*, *Veillonella*, *Yersinia*.	➞		**Depleted (15 Genera):** *Acinetobacter*, *Aeromonas*, *Bacillus*, *Candidatus symbiothrix*, *Dysgonomonas*, *Enterobacter*, *Escherichia*, *Eubacterium*, *Plesiomonas*, *Porphyromonas*, *Proteiniphilum*, *Shigella*, *Streptococcus*, *Veillonella*, *Yersinia*.
			**Introduced (1 Genera):** *Pseudomonas*.

Using controlled ANOVA we evaluated whether any specific bacterial genera were significantly different between the source water and filtered water samples. There were relatively few genera that were significantly different between the groups, the most notable being *Klebsiella* (Table 2). The significantly low abundance of *Klebsiella* in POU water as compared to corresponding source waters may indicate reduction of the overall coliform load of POU water which is primarily used for drinking or cooking purposes.

**Table 2:** Genera listed are significantly different between the groups.

Bacterial genera	Category	Mean	Groups
*Klebsiella*	Source Water	30.429	A
	POU Water	12.314	B
*Kluyvera*	Source Water	0.686	A
	POU Water	0.125	B
*Citrobacter*	Source Water	0.374	A
	POU Water	0.107	B
*Shimwellia*	Source Water	0.066	A
	POU Water	0.019	B
*Morganella*	Source Water	0.013	A
	POU Water	0.000	B

These findings were consistent with previously observed health complaints of area residents for which diarrheal disease, gastrointestinal disorders, unexplained fevers, and respiratory tract infections (especially in children) are commonplace (unpublished data).

## Conclusions

In this study, the identification of bacterial communities in source and POU water samples was performed using pyrosequencing methods. The identification was determined by bacteria-specific nucleic acid markers regardless of viability status of the bacteria. These methodologies report non-viable or non-culturable cells along with culturable cells. Therefore, the results should be interpreted differently than those reported by growth-based and culture-dependent studies. A mere detection of the “presence” of certain bacteria in the water samples by sequencing-based methods (such as the present study) may not indicate the presence of live and active cells. Viability PCR (vPCR) or rRNA-based methods relying on detecting the synthesis of a species-specific rRNA precursor (pre-rRNA), RNAsec (metatranscriptomics), are required to confirm the presence of viable bacteria. In addition, functional information such as presence of virulence factors can be acquired by conducting real-time PCR-based assays. Nevertheless, the rich bacterial diversity and potential pathogenic organisms present in the water samples may represent a public health concern, and can serve as a great tool for risk assessment. The results of this study indicate the importance of ongoing water quality monitoring in rural Haiti supplemented with water sanitation and hygiene (WASH) education for the local population.

## Supporting Information

S1 Fig Map of study area. (TIF)

S2 Fig Relative abundance of bacterial diversity at the genus level in the source and point-of-use water samples. Bacterial genus abundance less than 1% were grouped as “Others”.
(TIF)

S1 Table Sample Description. (DOCX)
