# Supplementary figures and images for: Microbial Diversity of Source and Point-of-Use Water in Rural Haiti – A Pyrosequencing-Based Metagenomic Survey
Source: PLoS One. 2016 Dec 9;11(12):e0167353. doi: 10.1371/journal.pone.0167353 (PMC5147895; doi:10.1371/journal.pone.0167353)

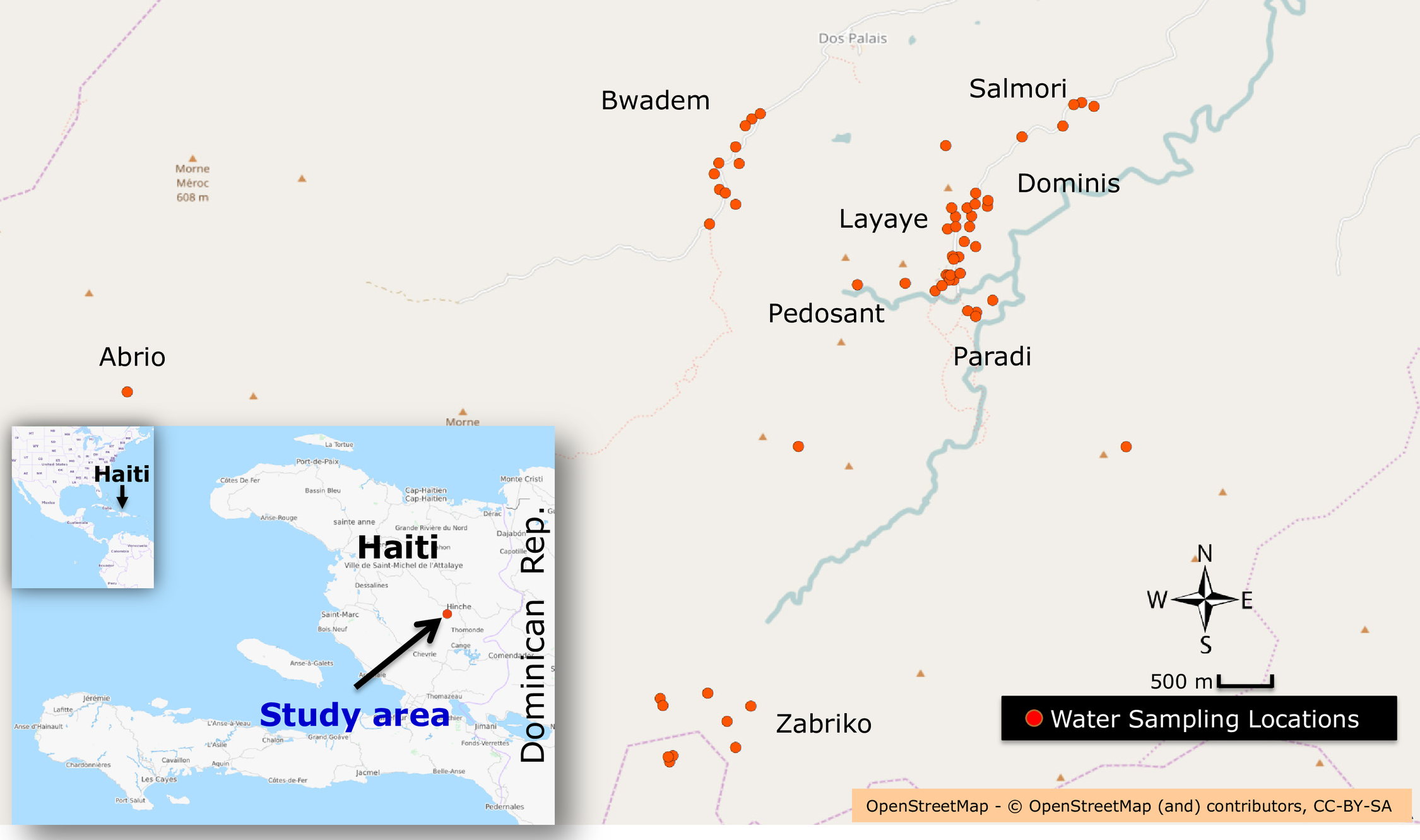

Supplement: S1 Fig — (TIF) [file pone.0167353.s001.tif]

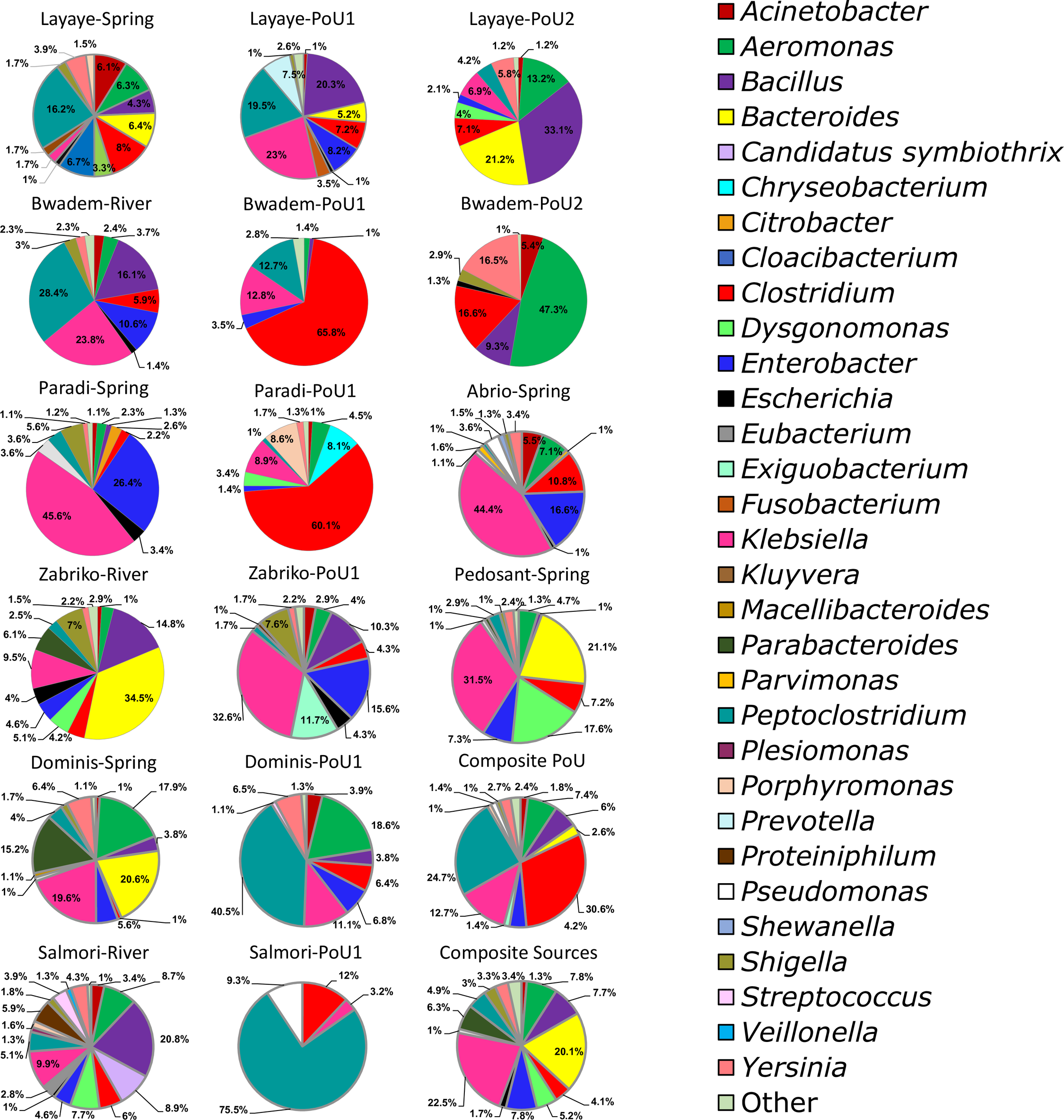

Supplement: S2 Fig — Bacterial genus abundance less than 1% were grouped as “Others”. (TIF) [file pone.0167353.s002.tif]
