# Supplementary material for: Microbial Diversity of Source and Point-of-Use Water in Rural Haiti – A Pyrosequencing-Based Metagenomic Survey
Source: PLoS One. 2016 Dec 9;11(12):e0167353. doi: 10.1371/journal.pone.0167353 (PMC5147895; doi:10.1371/journal.pone.0167353)
Supplement: S1 Table — (DOCX) [file pone.0167353.s003.docx]

**Supporting Table S1.** Sample Description.

| **Group** | **Sample description** | **Samples (n)** |
| --- | --- | --- |
|  | (village/water source) |  |
|  |  |  |
|  |  |  |
| Layaye-Spring | Layaye- major water source (springs) | 10 |
|  |  |  |
| Layaye-POU1 | Point-of-use | 12 |
|  | Layaye/two bucket filter |  |
|  |  |  |
| Layaye-POU2 | Point-of-use | 6 |
|  | Layaye/biosand filter |  |
|  |  |  |
| Bwadem-River | Bwadem-major water source (river) | 10 |
|  |  |  |
| Bwadem-POU1 | Point-of-use | 7 |
|  | Bwadem/two bucket filter |  |
|  |  |  |
| Bwadem-POU2 | Point-of-use | 1 |
|  | Bwadem/biosand filter |  |
|  |  |  |
| Paradi-Spring | Paradi- major water source (Belo spring) | 1 |
|  |  |  |
| Paradi-POU1 | Point-of-use | 3 |
|  | Paradi/two bucket filter |  |
|  |  |  |
| Zabriko-River | Zabriko- major source (spring, Zabriko river) | 9 |
|  |  |  |
| Zabriko-POU1 | Point-of-use | 10 |
|  | Zabriko/Sawyer filter |  |
|  |  |  |
| Abrio-Spring | Abrio- major source (spring) | 2 |
|  |  |  |
| Pedosant-Spring | Pedosant - major water source (spring) | 5 |
|  |  |  |
| Dominis-Spring | Dominis - major water source (spring) | 5 |
|  |  |  |
| Dominis-POU1 | Point-of-use | 9 |
|  | Dominis/ two bucket filter |  |
|  |  |  |
| Salmori-River | Salmori - major water source (river) | 4 |
|  |  |  |
| Salmori-POU1 | Point-of-use | 1 |
|  | Salmori/two bucket filter |  |
| Composite-POU | Composite of all POUs (of all 8 villages) |  |
| Composite-Sources | Composite of all water sources |  |
|  |  |  |
